# Effect of High-dose Antithrombin Supplementation in Patients with Septic Shock and Disseminated Intravascular Coagulation

**DOI:** 10.1038/s41598-019-52968-y

**Published:** 2019-11-12

**Authors:** Youn-Jung Kim, Byuk Sung Ko, Seo Young Park, Dong Kyu Oh, Sang-Bum Hong, Seongsoo Jang, Won Young Kim

**Affiliations:** 1 Department of Emergency Medicine, Asan Medical Center, University of Ulsan College of Medicine, 88 Olympic-ro 43-gil, Songpa-gu, Seoul 05505 Korea; 2 Department of Emergency Medicine, College of Medicine, Hanyang University, 222 Wangsimni-ro, Seongdong-gu, Seoul 133-791 Korea; 3 Department of Clinical Epidemiology and Biostatistics, Asan Medical Center, University of Ulsan College of Medicine, 88 Olympic-ro 43-gil, Songpa-gu, Seoul 05505 Korea; 4 Department of Pulmonary and Critical Medicine, Asan Medical Center, University of Ulsan College of Medicine, 88 Olympic-ro 43-gil, Songpa-gu, Seoul 05505 Korea; 5 Department of Laboratory Medicine, Asan Medical Center, University of Ulsan College of Medicine, 88 Olympic-ro 43-gil, Songpa-gu, Seoul 05505 Korea

**Keywords:** Infectious diseases, Combination drug therapy

## Abstract

The efficacy of antithrombin (AT) administration in patients with septic shock and disseminated intravascular coagulation (DIC) was uncertain. This study aimed to investigate whether high-dose AT administration improves outcomes in patients with septic shock and DIC. This observational, prospective cohort study included consecutive adult septic shock patients with DIC who showed AT activity <70% between March 2016 and August 2018. The 28 day mortality of the patients treated with AT and without AT was evaluated by propensity score matching and inverse probability of treatment weighting. Among 142 patients with septic shock and DIC, 45 patients (31.7%) received AT supplementation and 97 did not. The 28 day mortality rate was lower in the AT group, but no statistically significant difference persisted after matching. Multivariable analysis showed that AT supplementation was independently associated with 28 day mortality (odds ratio [OR], 0.342; 95% CI [confidence interval], 0.133−0.876; P = 0.025); however, no such association was observed after matching (OR, 0.480; 95% CI, 0.177−1.301; P = 0.149). High-dose AT administration in septic shock patients with DIC showed the improvement in survival, but the improvement was not observed after matching. Further larger studies are needed to conclusively confirm these findings.

## Introduction

Sepsis is a heterogenous syndrome, defined as life-threatening organ dysfunction caused by a dysregulated host response to infection, and sepsis and septic shock represent a major global health problem^1,2^. Despite recent improvements in the understanding of the pathophysiology of sepsis and advances in clinical management, patient outcomes remain poor and have not significantly improved^3^. Currently, early, appropriate administration of antibiotics, fluid, and vasopressors are the only pharmacologic treatments shown to be associated with a survival benefit in these patients^1^. Additional adjuvant therapies are therefore required to optimize treatment and to individualize patient management according to their accompanying organ dysfunction.

Disseminated intravascular coagulation (DIC) is a frequent complication of septic shock and is associated with a high level of mortality^1,4,5^. In patients with septic shock, an impaired physiologic anticoagulant mechanism, particularly decreased antithrombin (AT) activity, leads to excessive microthrombus formation, microcirculatory dysfunction and, therefore, organ dysfunction^6^. AT is an important physiologic anticoagulant that affects the intrinsic, extrinsic, and common coagulation pathways, as well as exerting anti-inflammatory effects^7,8^. Despite the theoretical benefits of AT supplementation, current clinical practice guidelines do not support the use of AT in patients with severe sepsis and septic shock. These recommendations are based on data from the Phase III KyberSept clinical trial, which demonstrated that AT supplementation had no beneficial effect on overall mortality and that treatment was associated with an increased risk of bleeding^1,8^. However, a post hoc analysis and systematic reviews have shown the possible benefit of AT in specific subgroups of patients, such as those with severe sepsis with DIC who did not receive concomitant heparin^9–11^. Uncertainty surrounding the efficacy of AT supplementation in these patients has resulted in inconsistency in clinical practice^12^. Therefore, this prospective cohort study aimed to investigate whether high-dose AT administration improves the clinical outcomes of patients with septic shock associated with DIC during the early phase of the condition.

## Results

**Figure 1:**
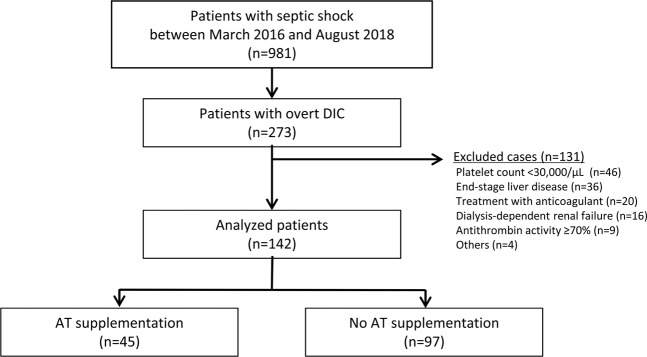
Patient flow diagram. Abbreviations: DIC, disseminated intravascular coagulation.

From a total of 981 patients with septic shock treated between March 2016 and August 2018, 273 were diagnosed with overt DIC (International Society on Thrombosis and Hemostasis [ISTH] score ≥5) and were enrolled in this study; 131 patients who were not indicated for AT supplementation were excluded. Therefore, a total of 142 patients were included in the analysis; 45 patients received AT supplementation and 97 patients did not (Fig. 1). Propensity score matching was performed in 68 patients (N = 34 in each group).

The baseline and clinical characteristics did not differ significantly between the AT supplementation and no AT supplementation groups (Table 1). The median patient age was 67.5 years (interquartile ranges [IQR], 59.0–76.0), and 87 patients (61.3%) were male. The most common infection focus was that of the hepatobiliary system (46.5%), followed by respiratory infection (16.9%). The median maximum sequential organ failure assessment (SOFA) and acute physiology and chronic health evaluation (APACHE) II scores were 10.0 (IQR, 7.8–12.0) and 20.0 (IQR, 14.8–24.3), respectively. Two thirds (66.2%) of the study patients showed overt DIC at ED presentation. The median DIC score was higher in the AT supplementation group than the no AT supplementation group, although this difference did not reach statistical significance (median, 6.0 vs. 5.0, P = 0.051). The baseline and clinical characteristics of the study patients after propensity matching and inverse probability of treatment weighting (IPTW) are presented in Supplementary Tables 2 and 3, respectively.

**Table 1:** Baseline and clinical characteristics of the study patients.

Characteristics	Total n = 142	AT supplementation n = 45	No AT supplementation n = 97	P-value
**Comorbid disease**				
Age, years	67.5 (59.0–76.0)	67.0 (54.0–76.0)	68.0 (60.0–76.5)	0.588
Male	87 (61.3)	22 (48.9)	65 (67.0)	0.039
Hypertension	50 (35.2)	16 (35.6)	34 (35.1)	0.953
Diabetes mellitus	31 (21.8)	8 (17.8)	23 (23.7)	0.426
Metastatic solid cancer	72 (50.7)	22 (48.9)	50 (51.5)	0.768
Other comorbid disease^a^	7 (4.9)	0 (0)	7 (7.2)	0.097
Infection focus				0.105
Respiratory system	24 (16.9)	4 (8.9)	20 (20.6)	
Hepatobiliary system	66 (46.5)	26 (57.8)	40 (41.2)	
Others	52 (36.6)	15 (33.3)	37 (38.1)	
**Laboratory findings**				
White blood cell count, /µL	8050 (4150–17825)	8300 (4850–15850)	7700 (3500–18250)	0.585
Hemoglobin, g/dL	10.6 (2.15)	10.6 (2.36)	10.6 (2.05)	0.966
Platelet, ×10^3^/µL	81.0 (47.8–129.8)	78.0 (46.0–142.0)	85.0 (51.0–128.5)	0.488
Prothrombin time, INR	1.53 (1.35–1.74)	1.56 (1.42–1.72)	1.51 (1.34–1.78)	0.593
Sodium, mmol/L	133.9 (5.87)	133.9 (5.03)	134.0 (6.25)	0.923
Potassium, mmol/L	4.2 (3.6–4.7)	4.1 (3.5–4.7)	4.2 (3.6–4.8)	0.266
Chloride, mmol/L	98.5 (6.62)	98.6 (6.52)	98.5 (6.71)	0.950
Creatinine, mg/dL	1.49 (1.07–2.29)	1.20 (1.02–2.29)	1.55 (1.17–2.31)	0.066
Albumin, g/dL	2.4 (2.0–2.7)	2.3 (2.1–2.8)	2.4 (2.0–2.7)	0.918
CRP, mg/dL	15.00 (6.48–21.96)	14.48 (5.22–21.75)	15.89 (6.59–22.78)	0.535
Lactic acid, mmol/L	4.7 (2.9–7.4)	4.2 (2.5–6.4)	4.9 (3.2–7.7)	0.112
**Severity score**				
SOFA score	10.0 (7.8–12.0)	9.0 (7.0–12.0)	10.0 (8.0–12.0)	0.201
APACHE II score	20.0 (14.8–24.3)	20.0 (13.5–25.5)	20.0 (16.0–24.0)	0.787
DIC documentation				0.399
At presentation	94 (66.2)	32 (71.1)	62 (63.9)	
<24 hours after admission	48 (33.8)	13 (28.9)	35 (36.1)	
DIC score by ISTH criteria	5.0 (5.0–6.0)	6.0 (5.0–6.0)	5.0 (5.0–6.0)	0.051
Antithrombin level, %	45.5 (34.0–55.0)	44.0 (35.0–53.5)	46.0 (33.0–55.0)	0.550

Data are shown as median (interquartile range) or as n (%).

^a^Other comorbid disease includes coronary artery disease, chronic pulmonary disease, liver cirrhosis, chronic kidney disease, and previous cerebrovascular accident.

Abbreviations: APACHE, acute physiology and chronic health evaluation; AT, antithrombin; CRP, C-reactive protein; DIC, disseminated intravascular coagulation; INR, international normalized ratio; ISTH, International Society on Thrombosis and Hemostasis; SOFA, sequential organ failure assessment.

Table 2 compares the clinical outcomes before and after propensity score matching. Prior to matching, the 28 day mortality was significantly lower in the AT supplementation group than the no AT supplementation group (17.8% vs. 34.0%, P = 0.047), while other secondary clinical outcomes showed no statistically significant differences. In the propensity-matched cohort, all clinical outcomes (including 28 and 90 day mortality, recovery of organ function, and changes in SOFA score) showed no statistically significant differences between the two groups. No major bleeding events were seen in either group for 7 days.

**Table 2:** Comparisons of Clinical Outcomes.

Clinical Outcomes	Overall cohort			Propensity-matched cohort		
	AT supplementation n = 45	No AT supplementation n = 97	P-value	AT supplementation n = 34	No AT supplementation n = 34	P-value
28 day mortality	8 (17.8)	33 (34.0)	0.047	8 (23.5)	9 (26.5)	0.786
90 day mortality	16 (35.6)	45 (48.4)	0.155	14 (41.2)	14 (41.2)	0.754
Recovery of organ function	35 (77.8)	68 (70.1)	0.340	26 (76.5)	27 (79.4)	0.777
Changes in SOFA score	4.0 (2.0–7.0)	4.0 (0.0–7.0)	0.342	4.0 (2.0–7.0)	5.0 (2.0–7.8)	0.317

Data are shown as median (interquartile range) or as n (%).

Abbreviations: AT, antithrombin; SOFA, sequential organ failure assessment.

**Figure 2:**
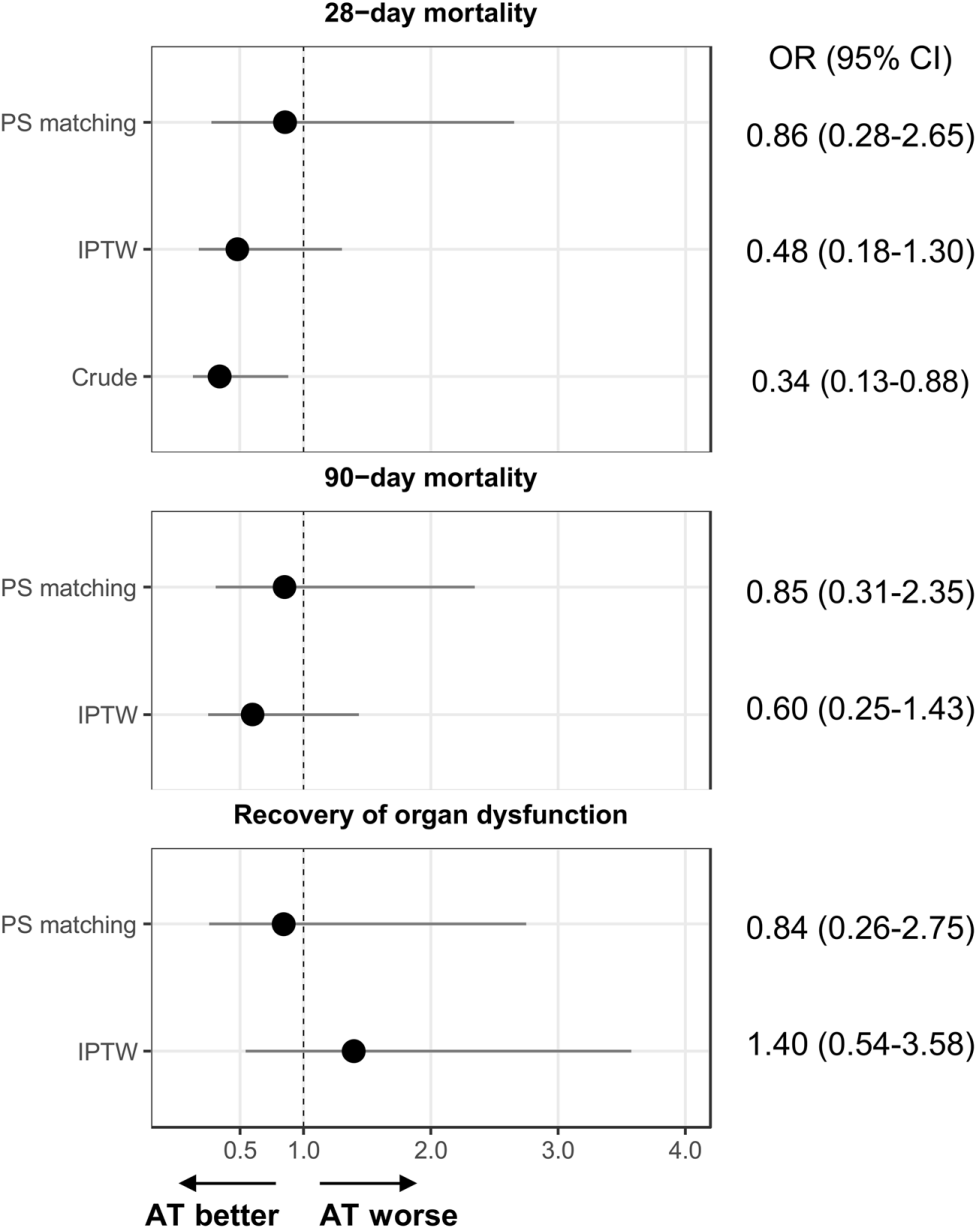
Odds ratios for clinical outcomes for antithrombin supplementation. Abbreviations: AT, antithrombin; CI, confidence interval; IPTW, inverse probability of treatment-weighted; PS, propensity score.

The odds ratios (ORs) of AT supplementation for clinical outcomes including 28 day mortality, 90 day mortality, and recovery of organ function in patients with septic shock and DIC are presented in Fig. 2. The logistic regression analysis of crude, propensity score-matched, and IPTW propensity data demonstrated that AT supplementation was not significantly associated with any of the clinical outcomes. In the overall cohort, AT supplementation significantly improved 28 day mortality (OR, 0.34; 95% CI, 0.13–0.88; P = 0.03). This tendency towards reduced 28 day mortality in patients with AT supplementation was still observed in propensity score-matched groups (OR, 0.69; 95% CI, 0.21–2.22; P = 0.531) and IPTW propensity data (OR, 0.42; 95% CI, 0.18–1.30; P = 0.149), respectively, although without reaching statistical significance.

## Discussion

In this prospective observational study, propensity score matching among the patients with septic shock and DIC who showed AT activity <70% showed no significant difference in 28 day mortality between patients receiving AT supplementation and those who did not. Despite the theoretical benefits of AT supplementation for patients with septic shock, this approach did not result in an improvement in clinical outcomes with respect to mortality and the recovery of organ function after propensity score analyses (matching and IPTW). However, patients who received AT supplementation had no serious bleeding events. Despite the low statistical power, this study provides information that can serve as a basis for clinical equipoise to support the need of further investigation and possibly randomized clinical trial.

Although DIC frequently accompanies sepsis and is associated with a high rate of mortality, the main therapeutic strategy remains to treat the underlying causative factor without other adjunct treatment^5^. AT supplementation has been suggested as a potential adjunct for the treatment of sepsis-associated DIC on the basis of post hoc analysis of the KyberSept trial and retrospective observational studies^8,10,13,14^. In addition, a recent multicenter retrospective observational study by Hayakawa *et al*. has suggested that AT supplementation for patients with sepsis-induced DIC may reduce the rate of in-hospital mortality^12^. However, these data were not statistically robust, with only IPTW propensity score analysis showing a significant association between AT supplementation and low in-hospital mortality (OR, 0.75; 95% CI, 0.57–0.98; P = 0.03); by contrast, a non-significant association was seen using quintile-stratified propensity score analysis and propensity score matching analysis^12^. Due to these conflicting results, the beneficial effect of AT supplementation on survival in patients with sepsis and DIC remains controversial.

In the current study, we aimed to evaluate the beneficial effect of AT supplementation in patients with septic shock and overt DIC who showed AT activity <70%. Based on the results of previous studies, this target group was considered to be most likely to benefit from adjunct treatment^8,9,11,12,14^. In a crude analysis, patients treated with or without AT supplementation showed no statistically significant differences in baseline and clinical characteristics. With respect to mortality, the crude 28 day mortality of the patient cohort was 28.9% (41/142), which is lower than that reported in previous studies of patients with severe sepsis and DIC, and those with septic shock and DIC, which have shown a 28 day mortality rate of 30–70%^4,14^. In the current study, there was an absolute difference of 14.2 percentage points in the crude model in favor of AT supplementation versus no AT supplementation, with the AT supplementation group showing a higher survival rate than those without (34.0 vs 17.8%, P = 0.047). When a randomized controlled trial is not possible, propensity score matching and IPTW analysis is a powerful approach to estimate treatment effect in observational studies, reducing treatment selection bias and the inherent limitations of an observational study^15^. After matching was carried out in the current study, AT supplementation in patients with septic shock and DIC showed a consistent tendency towards reducing mortality but the statistical significance of AT supplementation was diminished. This may have been due to the reduced number of study patients in the matched group. Decreased AT activity is another important prognostic factor in patients with sepsis and DIC, and AT supplementation has been seen to have a greater beneficial effect in patients with decreased AT activity, particularly in those with values <40%^14,16^. Hayakawa *et al*. recently suggested that patients with sepsis and DIC who had very low AT activity (<43%) were ideal candidates for AT supplementation, rather than those with AT activity <70%^17^. In the current study, the median AT activity of our study patients was 45.5%, and this relatively high level of AT activity may have contributed to the diminished beneficial effect of AT supplementation.

Our study has several limitations. First, it was conducted in a single center, which limits the generalizability of the results. Secondly, the small sample size could mean that the statistical analyses are underpowered. The relatively lower 28 day mortality rate and the limited sample size would contribute the lack of power, and might have contributed to the lack in significance of the results. Larger studies are needed to conclusively confirm these findings. The study design did not include the recovery of DIC or elevation of AT activity as secondary outcomes, but instead included a patient-centered outcome, 28 day mortality, as the primary outcome. Although recovery of DIC or improvement of AT activity would be important clinical outcomes, mortality is more clinically relevant. The study collected data only on major bleeding events, which were defined as intracranial or those requiring a transfusion of at least three units of blood. Although this approach did not collect data on minor bleeding events and consequently reduces the understanding of adverse events, it was considered to be a less subjective approach that could reduce diagnostic errors and ascertainment bias. In addition, although the attending physicians treated the patients according to the established guidelines, interventions such as the administration of appropriate antibiotics and low-dose hydrocortisone, the timing of administration of antibiotics, continuous renal replacement therapy and mechanical ventilation or other blinded variables could affect the clinical outcomes and be potential confounding factors.

In conclusion, AT supplementation for patients with septic shock and overt DIC who showed AT activity <70% was associated with improved survival with no increased risk of major bleeding event. However, this association was not seen after propensity matching and IPTW. Considering the heterogeneity of septic shock, larger studies are needed to conclusively confirm these findings and to identify candidates most likely to benefit from AT supplementation.

## Materials and Methods

### Study design and patient selection

This observational, prospective, cohort study of adult patients with septic shock and DIC was conducted in the emergency department (ED) of a university-affiliated, tertiary hospital in Seoul, Korea between March 2016 and August 2018. The septic shock registry has prospectively collected data for all consecutive adult patients (aged ≥19 years) with septic shock at EDs since June 2012^18^. Patients with septic shock were defined as those with refractory hypotension (systolic blood pressure <90 mmHg, mean arterial pressure <70 mmHg, or a systolic blood pressure decrease >40 mmHg) requiring vasopressor treatment despite receiving at least 20–30 mL/kg intravenous crystalloid, or those with hypoperfusion (blood lactate concentration ≥4 mmol/L) with suspected or confirmed infection^19,20^. The septic shock registry did not include patients assigned as ‘do not attempt resuscitation’, those transferred from other hospitals after treatment for septic shock, patients transferred directly from the ED to other hospitals, or those developing septic shock 6 hours after ED presentation.

Data from patients admitted with a diagnosis of septic shock and DIC were included in the analysis. Additional blood coagulation tests, including fibrinogen and fibrinogen degradation products, were routinely performed after November 2014^4^, and the measurement of AT activity level has been performed in patients with septic shock between 9 a.m. and 5 p.m. on weekdays since March 2016. DIC was defined in accordance with the criteria outlined by the ISTH (Supplementary Table 1), i.e., an ISTH DIC score ≥5^21^. Patients with an ISTH DIC score ≥5 and documented AT activity <70% were treated with AT (30 IU/kg for the first day and 3000 IU/day for the next two consecutive days) within 6 hours of diagnosis. Patients who met any of the following criteria were not treated with AT and were excluded from the study: AT activity ≥70%; evidence of bleeding; treatment with heparin (except subcutaneous low dose or intravenous line flushing) or oral anticoagulants; a platelet count <30,000/μL; immunocompromised status; acute myocardial infarction (within the previous 7 days); preexisting dialysis-dependent renal failure; end-stage liver disease; transplantation (postoperative state); history of stroke within the last year; history of hypersensitivity to AT; third-degree burns (≥20% of the total body area); and pregnancy^8^. Patients were categorized into AT supplementation or no AT supplementation groups. Patients in the no AT supplementation group comprised those individuals who could not undergo laboratory testing for AT activity within 6 hours of septic shock and DIC recognition as this facility was unavailable.

The study design was approved by the institutional review board of Asan Medical Center and informed consent was obtained prior to data collection (study no: 2016-0205). All procedures performed in this study were in accordance with the ethical standards of the institutional research committee and with the 1964 Helsinki Declaration and its later amendments or comparable ethical standards.

### Management and data collection

All patients with septic shock received treatment in accordance with the current 6 hour bundles of survival sepsis campaign, including the administration of intravenous crystalloid, obtaining blood cultures prior to the administration of antibiotics and the administration of broad-spectrum antibiotics and vasopressors^19^. Transfusion, including fresh frozen plasma or platelets, was not performed in the absence of significant bleeding, significant risk of bleeding, or planned invasive procedures^19^. Unfractionated or low molecular weight heparin was used to treat venous thrombosis and heparin was used for intravenous line flushing. The decision to include further interventions, such as mechanical ventilation and continuous renal replacement therapy, was based on standard institutional intensive care protocols under the direction of the attending intensive care physician.

The demographic and clinical characteristics of the patients (including age, sex, comorbid disease, focus of infection, laboratory findings, and severity scores such as SOFA and APACHE II scores) and clinical outcomes (such as 28 day mortality and in-hospital mortality) were retrieved from the septic shock registry. SOFA and APACHE II scores were calculated using the worst parameters within 24 hours of ED admission^22,23^. Recovery of organ function was defined as a decrease in SOFA score ≥2 from the first day to 7 days after ED presentation. ISTH scores and AT level were also collected. Major bleeding events, defined as intracranial or those requiring transfusion of ≥3 units of blood, were recorded for 7 days^8^.

The primary outcome of the study was 28 day mortality. Secondary outcomes were 90 day mortality, recovery of organ function within 7 days, and changes in SOFA score for 7 days after admission or until in-hospital death.

### Statistical analyses

Descriptive statistics (percentages; medians and IQR) were used to present participant characteristics overall, with chi-square tests or the Mann-Whitney U-test used to assess differences in patient characteristics between the AT supplementation and no AT supplementation groups. Because there were systematic differences between the two groups, two methods were considered to remove the confounding effect from the AT supplementation effect: propensity score matching and IPTW. These methods provide unbiased estimates of the treatment effect by creating a pseudo-population in which the treatment is like-randomized^24–26^. First, the propensity score (probability of receiving AT supplementation) was estimated for each patient by logistic regression using all covariates shown in Table 1. Patients who had similar propensity scores were matched in a 1:1 ratio, with caliper of 0.2 times the standard deviation of the logit of the propensity score, resulting in 34 propensity score-matched pairs. For IPTW, we assigned each patient a stabilized weight, which was the product of the marginal probability of receiving AT supplementation and the inverse of the propensity score for patients in the AT supplementation group. For patients in the no AT supplementation group, the stabilized weight was the product of the marginal probability of not receiving AT supplementation and the inverse of (1-propensity score). Propensity score-matched data and the data with IPTW were checked for group comparability using the standardized mean difference.

The effect of AT supplementation was evaluated using three methods: 1. crude multivariable logistic analysis, 2. analysis of propensity score-matched data, and 3. analysis of data with IPTW. In the crude analysis, multivariable logistic regression was conducted on the original data and was interpreted as the association between AT supplementation and outcomes. In the analyses of propensity score-matched or weighted data, logistic or linear regression was performed and the resulting OR estimates were interpreted as a closer approximation to the causal effect of the AT supplementation. All statistical analyses were performed using R 3.5.1 (R Foundation for Statistical Computing, Vienna, Austria).

## Supplementary information

Supplementary Tables

## Data Availability

The datasets used and/or analysed during the current study are available from the corresponding author on reasonable request.
