# Supplementary material for: Effect of High-dose Antithrombin Supplementation in Patients with Septic Shock and Disseminated Intravascular Coagulation
Source: Sci Rep. 2019 Nov 12;9:16626. doi: 10.1038/s41598-019-52968-y (PMC6851090; doi:10.1038/s41598-019-52968-y)
Supplement: Supplementary file 1 — Supplementary Tables [file 41598_2019_52968_MOESM1_ESM.docx]

**Effect of High-dose Antithrombin Supplementation in Patients with Septic Shock and Disseminated Intravascular Coagulation**

Youn-Jung Kim^1^; Byuk Sung Ko^2^; Seo Young Park^3^; Dong Kyu Oh^4^; Sang-Bum Hong^4^; Seongsoo Jang^5^; Won Young Kim^1, *^

^1^Department of Emergency Medicine, Asan Medical Center, University of Ulsan College of Medicine, 88 Olympic-ro 43-gil, Songpa-gu, Seoul 05505, Korea

^2^Department of Emergency Medicine, College of Medicine, Hanyang University, 222 Wangsimni-ro, Seongdong-gu, Seoul 133-791, Korea

^3^ Department of Clinical Epidemiology and Biostatistics, Asan Medical Center, University of Ulsan College of Medicine, 88 Olympic-ro 43-gil, Songpa-gu, Seoul 05505, Korea

^4^ Department of Pulmonary and Critical Medicine, Asan Medical Center, University of Ulsan College of Medicine, 88 Olympic-ro 43-gil, Songpa-gu, Seoul 05505, Korea

^5^ Department of Laboratory Medicine, Asan Medical Center, University of Ulsan College of Medicine, 88 Olympic-ro 43-gil, Songpa-gu, Seoul 05505, Korea

***Corresponding author:** Won Young Kim

Department of Emergency Medicine, University of Ulsan College of Medicine, Asan Medical Center, 88 Olympic-ro 43-gil, Songpa-gu, Seoul 05505, Korea

Tel.: +82-2-3010-3350; Fax: +82-2-3010-3360; E-mail: wonpia73@naver.com

**Running Title:** Antithrombin for septic shock with DIC

**Supplementary Table 1.** International Society on Thrombosis and Hemostasis Disseminated Intravascular Coagulation Scoring System

| Variable | Value | Points |
| --- | --- | --- |
| Platelet, ×10^3^/µL | > 100 | 0 |
|  | 50–100 | 1 |
|  | < 50 | 2 |
| Prolonged prothrombin time | < 3 seconds | 0 |
|  | 3–6 seconds | 1 |
|  | > 6 seconds | 2 |
| Fibrinogen, mg/dL | > 100 | 0 |
|  | < 100 | 1 |
| Elevated fibrin marker  (D-dimer or fibrin degradation products) | No increase | 0 |
|  | Moderate increase | 2 |
|  | Marked increase | 3 |

**Supplementary Table 2.** Baseline and clinical characteristics for the study patients after propensity matching analysis.

| Characteristics | AT supplementation n = 34 | No AT supplementation  n = 34 | Standardized difference of means |
| --- | --- | --- | --- |
| Age, years | 71.5 (62.3−76.8) | 65.0 (58.3−76.0) | 0.066 |
| Male | 20 (58.8) | 19 (55.9) | 0.447 |
| Comorbid disease |  |  |  |
| Hypertension | 12 (35.3) | 13 (38.2) | 0.061 |
| Diabetes mellitus | 8 (23.5) | 6 (17.6) | 0.146 |
| Metastatic solid cancer | 17 (50.0) | 16 (47.1) | 0.059 |
| Other comorbid disease *^a^* | 13 (38.2) | 11 (32.4) | 0.123 |
| Infection focus |  |  | 0.130 |
| Respiratory | 4 (11.8) | 4 (11.8) |  |
| Hepatobiliary and pancreatic | 18 (52.9) | 20 (58.8) |  |
| Others | 12 (35.3) | 10 (29.4) |  |
| Laboratory findings |  |  |  |
| White blood cell count, /µL | 10400 (5400−16125) | 8550 (5250−19675) | 0.148 |
| Hemoglobin, g/dL | 10.7 (2.17) | 10.7 (2.10) | 0.001 |
| Platelet, ×10^3^/µL | 84.0 (48.8−159.8) | 75.5 (45.0−128.8) | 0.119 |
| Prothrombin time, INR | 1.56 (1.41−1.69) | 1.54 (1.34−1.81) | 0.073 |
| Sodium, mmol/L | 134.1 (4.75) | 133.7 (6.71) | 0.066 |
| Potassium, mmol/L | 4.2 (3.7−4.8) | 4.0 (3.5−4.4) | 0.242 |
| Chloride, mmol/L | 98.6 (6.81) | 98.6 (7.30) | < 0.001 |
| Creatinine, mg/dL | 1.43 (1.07−2.33) | 1.48 (1.14−1.92) | 0.055 |
| Albumin, g/dL | 2.4 (2.1−2.9) | 2.4 (1.9−2.5) | 0.280 |
| CRP, mg/dL | 12.12 (4.61−19.60) | 15.32 (5.73−20.66) | 0.048 |
| Lactic acid, mmol/L | 5.2 (3.3−7.1) | 4.2 (2.4−5.8) | 0.172 |
| APACHE II score | 19.0 (14.0−24.8) | 19.0 (14.3−23.0) | 0.170 |
| DIC documentation |  |  | 0.061 |
| At presentation | 22 (64.7) | 21 (61.8) |  |
| < 24 hours after admission | 12 (35.3) | 13 (38.2) |  |
| DIC score by ISTH criteria | 6.0 (5.0−6.0) | 5.0 (5.0−6.0) | 0.104 |
| Antithrombin level, % | 44.5 (35.0−51.8) | 41.5 (30.0–52.0) | 0.123 |

Data are shown as mean (standard deviation), median (interquartile range), or n (%).

*^a^* Other comorbid disease includes coronary artery disease, chronic pulmonary disease, liver cirrhosis, chronic kidney disease, and previous cerebrovascular accident.

Abbreviations: APACHE, acute physiology and chronic health evaluation; AT, antithrombin; CRP, C-reactive protein; DIC, disseminated intravascular coagulation; INR, international normalized ratio; ISTH, International Society on Thrombosis and Hemostasis

**Supplementary Table 3.** The baseline and clinical characteristics for the study patients after inverse probability of treatment weighting analysis.

| Characteristics | AT supplementation | No AT supplementation | Standardized difference of means |
| --- | --- | --- | --- |
| Age, years | 69.0 (62.2−76.0) | 67.6 (59.0−76.0) | 0.024 |
| Male | 25.4 (55.9) | 57.9 (62.0) | 0.123 |
| Comorbid disease |  |  |  |
| Hypertension | 18.2 (40.1) | 34.1 (36.5) | 0.073 |
| Diabetes mellitus | 8.7 (29.2) | 21.0 (22.5) | 0.084 |
| Metastatic solid cancer | 22.8 (50.2) | 48.0 (51.4) | 0.025 |
| Other comorbid disease *^a^* | 20.8 (45.9) | 40.7 (43.6) | 0.046 |
| Infection focus |  |  | 0.155 |
| Respiratory | 5.7 (12.6) | 16.4 (17.6) |  |
| Hepatobiliary and pancreatic | 20.2 (44.4) | 42.0 (44.9) |  |
| Others | 19.5 (42.9) | 35.0 (37.5) |  |
| Laboratory findings |  |  |  |
| White blood cell count, /µL | 6655 (4578−15044) | 7762 (3766−18297) | 0.056 |
| Hemoglobin, g/dL | 10.8 (2.22) | 10.7 (2.06) | 0.085 |
| Platelet, ×10^3^/µL | 68.7 (45.5−102.0) | 85.7 (47.0−128.1) | 0.104 |
| Prothrombin time, INR | 1.55 (1.41−1.68) | 1.53 (1.34−1.78) | 0.089 |
| Sodium, mmol/L | 134.0 (4.57) | 134.1 (6.48) | 0.009 |
| Potassium, mmol/L | 4.0 (3.5−4.7) | 4.1 (3.6−4.7) | 0.133 |
| Chloride, mmol/L | 98.5 (6.04) | 98.6 (6.89) | 0.012 |
| Creatinine, mg/dL | 1.38 (1.02−2.32) | 1.48 (1.05−2.18) | 0.152 |
| Albumin, g/dL | 2.3 (2.1−3.0) | 2.4 (2.0−2.7) | 0.057 |
| CRP, mg/dL | 12.95 (3.57−19.63) | 13.14 (6.15−21.09) | 0.113 |
| Lactic acid, mmol/L | 5.1 (2.5−7.1) | 4.7 (3.0−7.5) | 0.120 |
| APACHE II score | 18.5 (13.0−23.2) | 20.0 (16.0−24.0) | 0.064 |
| DIC documentation |  |  | 0.064 |
| < 24 hours after admission | 16.0 (35.4) | 33.4 (35.8) |  |
| DIC score by ISTH criteria | 5.0 (5.0−6.0) | 5.0 (5.0−6.0) | 0.036 |
| Antithrombin level | 40.1 (31.6−53.6) | 45.3 (31.4−55.0) | 0.023 |

Data are shown as mean (standard deviation), median (interquartile range), or n (%).

*^a^* Other comorbid disease includes coronary artery disease, chronic pulmonary disease, liver cirrhosis, chronic kidney disease, and previous cerebrovascular accident.

Abbreviations: APACHE, acute physiology and chronic health evaluation; AT, antithrombin; CRP, C-reactive protein; DIC, disseminated intravascular coagulation; INR, international normalized ratio; ISTH, International Society on Thrombosis and Hemostasis.
